# Prognostic Value of Coronary Angiography-Derived Fractional Flow Reserve Immediately After Stenting

**DOI:** 10.3389/fcvm.2022.834553

**Published:** 2022-03-21

**Authors:** Zuoyi Zhou, Baozhen Zhu, Fangfang Fan, Fan Yang, Shu Fang, Zhi Wang, Lin Qiu, Yanjun Gong, Yong Huo

**Affiliations:** ^1^Department of Cardiology, Peking University First Hospital, Beijing, China; ^2^Department of Intervention, Tongxin People's Hospital, Tongxin, China

**Keywords:** coronary angiography-derived fractional flow reserve, percutaneous coronary intervention, drug-eluting stent, late lumen loss, target vessel failure

## Abstract

**Objectives:**

The aim of this study was to investigate the potential prognostic value of post-percutaneous coronary intervention (PCI) angiography-derived fractional flow reserve (FFR) and its gradient across the stent.

**Background:**

Post-PCI FFR and its gradient across the stent have been proved to be associated with clinical outcomes. However, little is known about the prognostic value of post-PCI coronary angiography-derived FFR and its gradient across the stent.

**Methods:**

Patients diagnosed with coronary heart disease and participated in drug-eluting stent (DES) clinical trials for stent implantation in a single center were included for this retrospective analysis. A novel coronary angiography-derived FFR (caFFR) and its gradient across the stent were calculated offline using two projections from coronary angiography performed after PCI. Clinical follow-up was completed at 9 months after the index procedure and the primary outcome was target vessel failure (TVF), defined as a composite of target vessel-related myocardial infarction (MI), target vessel-related revascularization (TVR), and cardiac death. Coronary angiography was also performed at the 9 months follow-up time to get data of late lumen loss (LLL) and percent diameter stenosis (%DS).

**Results:**

A total of 159 vessels in 136 patients were analyzed. The mean value of post-PCI caFFR was 0.90 ± 0.06. The median value of trans-stent caFFR gradient (ΔcaFFR_stent_) was 0.04 (interquartile range 0.02–0.08). ΔcaFFR_stent_>0 was demonstrated in 147 vessels (92.45%). The TVF rate was significantly higher in patients with post-PCI caFFR < 0.90 (4 [8.16%] vs. 1 [1.15%], *P* = 0.037), which was mainly achieved by the difference between the TVR rate. In the subgroup with lesions located in the left anterior descending coronary artery (LAD), post-PCI caFFR was an independent predictor of LLL (β = −1.07, 95% CI: −1.74 to −0.39, *P* = 0.002) and %DS at follow-up (β = −30.24, 95% CI: −56.44 to −4.04, *P* = 0.025), ΔcaFFR_stent_ was an independent predictor of LLL (β=0.98, 95% CI:0.13–1.83, *P* = 0.026).

**Conclusion:**

Suboptimal post-PCI caFFR and trans-stent caFFR gradient were common among vessels immediately after stenting. Lower post-PCI caFFR was associated with a higher rate of 9-month TVF. After LAD PCI, both post-PCI caFFR and its gradient across stent were independent predictors of the neointimal proliferation of the target vessel evaluated by LLL and %DS at follow-up.

## Introduction

Fractional flow reserve (FFR), first proposed by Pijls in 1996, is a reliable functional index that can recognize perfusion-affecting epicardial coronary lesions (1). Several clinical trials have confirmed the long-term prognostic benefit of using FFR assessment before percutaneous coronary intervention (PCI) procedures (2–4). Based on that evidence, European society of cardiology (ESC) recommended FFR assessment for intermediate stenosis (typically around 40–90%) when no evidence of ischemia is available (class I indication) (5). However, after angiographically satisfactory PCI, some patients still suffered major adverse cardiac event (MACE) or target vessel events at follow-up (6, 7). In a clinical trial designed to assess device-specific outcomes after implantation of different kinds of stents, MACE was recorded in more than 20% of the patients in a two-year follow-up after the procedure (6). These events may result from various reasons including the existence of untreated hemodynamically significant stenosis that can hardly be found by post-stenting angiography, under expansion of the stent, and/or the presence of diffuse disease (8). Better tools for evaluating post-PCI coronary physiology are consistently needed. FFR immediately measured after stenting seems a good choice. Recently, a couple of prospective and retrospective clinical studies have preliminarily shown the value of post-PCI FFR on determining persistent ischemia after intervention and predicting long-term events both in PCI with stent implantation (9–13) and drug-coated balloon only PCI (14, 15). The presence of residual major FFR gradient found in post-PCI FFR pull-back tracings was also found as an independent predictor for target vessel failure (TVF) at 2 years (16). What's more, for PCI with stent implantation, another study found that FFR gradient across the stent can also be a novel index to predict long-term clinical outcomes of patients. FFR gradient across the stent ≥ 0.04 and the FFR gradient across stent divided by the total stent length multiplied by 10 ≥ 0.009 predicted suboptimal stenting and were independent predictors of MACE in the 10-year follow up (17). Nevertheless, the application of post-PCI FFR assessment is still underutilized because of not only the technical and economic reasons but also the operator's reluctance to FFR assessment after an angiographically satisfactory PCI (18, 19). Thus, more simplified techniques are called for facilitating physicians to perform functional assessment after a long PCI and further increasing patients' prognosis. Coronary angiography-derived FFR is a new technique recently developed. Compared to the traditional pressure wire-based FFR, Coronary angiography-derived FFR does not need wire placement and is adenosine-free, the total operation times are also greatly shortened (20–23). Using invasive FFR as standard, a number of studies have confirmed the diagnostic accuracy of at least four kinds of angiography-derived FFR (23, 24), including one novel computational pressure-flow dynamics (CPFD) derived FFR (caFFR) developed by our team (23). Both caFFR and quantitative flow ratio (QFR) are angiography-based FFR computational products. On the other hand, caFFR was calculated with the optimized CFD method in the FLASH ANGIO software (Rainmed Ltd., Suzhou, China), using two projections of angiography images and patient-specific aortic pressures while QFR was computed with a simplified mathematical model with a fixed MAP. In the FLASH FFR study with 330 patients enrolled in six centers, caFFR showed high accuracy of 95.7% in comparison with the wire-based FFR measurements (23). However, the data of post-PCI non-invasive FFR and its relevance to patient prognosis are sparse (25, 26)and the data of trans-stent non-invasive FFR gradient has never been reported. The aim of this study is to preliminarily explore whether post-PCI caFFR and trans-stent caFFR gradient will have an impact on patients' prognosis.

## Methods

### Study Design and Patient Population

This study was a retrospective cohort study. Patients diagnosed with coronary artery disease with visually lumen diameter stenosis ≥ 70% by coronary angiography and participated in clinical trials of drug-eluting stent (DES) with DES implantation in the Department of Cardiology, Peking University First Hospital from April 2009 to June 2014 were included in this study if they are (1) ≥ 18 years old; (2) DESs were implanted in at least one major coronary vessel, including left anterior descending (LAD), left circumflex (LCX), or right coronary Artery (RCA); (3) postoperative follow-up data at 9 months were complete. The exclusion criteria contain: (1) prior stenting of the target vessel; (2) total occlusion of the target vessel; (3) coronary artery bypass grafting; (4) the target vessel provides collateral circulation to another vessel; (5) the obtained angiographic projections were not suitable for analysis. The study was performed following the Declaration of Helsinki, and the study was approved by the Ethics Committee of Peking University First Hospital.

### Study Procedure

Medical preparation, invasive coronary angiography, and PCI were performed following the practices of the department of cardiology of Peking University First Hospital. Whether to perform post-dilation was at the operators' discretion.

### Quantitative Coronary Angiography (QCA)

With optimal projections, the quantitative coronary analysis was performed by a validated software (CAAS 5.9.2, Pie Medical Imaging, The Netherlands). The reference lumen diameter, minimal lumen diameter (MLD), percent diameter stenosis (%DS), and lesion length were measured before and after PCI and at 9-month follow-up. Late lumen loss was calculated by the difference of MLD immediately after PCI and at 9-month follow-up (LLL = MLD immediately after PCI–MLD at 9-month follow-up). All these measurements were independently performed by two certified operators and intra-observer and inter-observer agreements were very high (ICC > 0.90).

### CaFFR

The generation of caFFR was performed as previously described (23). At least two angiographic projections separated by ≥ 30° were acquired for the calculation of caFFR using a validated software (FLASH ANGIO, Rainmed, China). The target vessels' caFFR before and after PCI were measured at the distal part of the target vessel. After stenting, the caFFR proximal to stent (p-caFFR) and distal to stent (d-caFFR) were recorded. The FLASH ANGIO software can show the value of the caFFR of any point along the meshed coronary arteries in the vessel path from the inlet to the most distal position. As long as the operator pinpoints the position of the inlet and the outlet of the stent, the p-caFFR and d-caFFR of the stent will be shown immediately. The ΔcaFFR_stent_ is defined as the difference between p-caFFR and d-caFFR (ΔcaFFR_stent_ = p-caFFR–d-caFFR) and the ΔcaFFR_stent/length_ is defined as ΔcaFFR_stent_ divided by stent length before multiple by 10 (ΔcaFFR_stent/length_ = [ΔcaFFR_stent_/stent length] × 10). All these measurements were independently performed by two certified operators and the intra-observer and inter-observer agreements were very high (ICC > 0.90).

### Data Collection, Follow-Up, and Study Endpoint

The participants' demographic data and cardiovascular risk factors were collected. The baseline levels of left ventricular ejection fraction (LVEF), low-density lipoprotein cholesterol (LDL-C), serum creatine (CREA), and type B natriuretic peptide (BNP) were also recorded at the time of PCI. Clinical follow-up and coronary angiography were performed at 9 months.

The primary endpoint was TVF, defined as a composite of cardiac death, target vessel-related myocardial infarction (MI), and target vessel revascularization (TVR). MI was defined according to the Fourth Universal Definition of MI (27). TVR is composed of PCI and coronary artery bypass graft (CABG) of the target vessels (28). Cardiac death was defined as death with a cardiac cause, such as malignant arrhythmia, heart failure, and MI.

### Statistical Analysis

Continuous variables were presented as mean ± SD with comparison by independent sample *t*-test for normal distribution and were reported as medians with interquartile range with a comparison by Kruskal-Wallis test for skewed distribution. Categorical variables were expressed as counts and percentages and the differences between groups were tested by chi-square test or Fisher's exact probability test (counts < 10). Intra- and inter-measurer agreements were tested using the intraclass correlation coefficient (ICC) analysis. According to the findings from studies of invasive post-PCI FFR, we chose 0.9 as the cut-off value of post-PCI caFFR (12, 17), 0.04 as the cut-off value of ΔcaFFR_stent_,. And 0.009 as the cut-off value of ΔcaFFR_stent/length_ (17) to classify the patients. The differences in the rates of events and the differences in QCA results at the follow-up time between the groups were tested for statistical significance. The predictive values of the post-PCI functional assessments of the target vessels for LLL and %DS at follow-up were analyzed using univariate and multivariate linear regression, with adjustment for age, sex, and diabetes mellitus. One-way logistic regression was used to analyze the predictive values of post-PCI functional indexes of the target vessels for TVF at follow-up. Subgroup tests were also performed based on the location of the stent (left anterior descending or not). All tests were two-sided, and *P* < 0.05 was considered statistically significant. All statistics in this study were performed using Empower (www.empowerstats.com) and R software (http://www.R-project.org).

## Results

### Characteristics of Patients, Lesions, and Study Procedures

**Figure 1:**
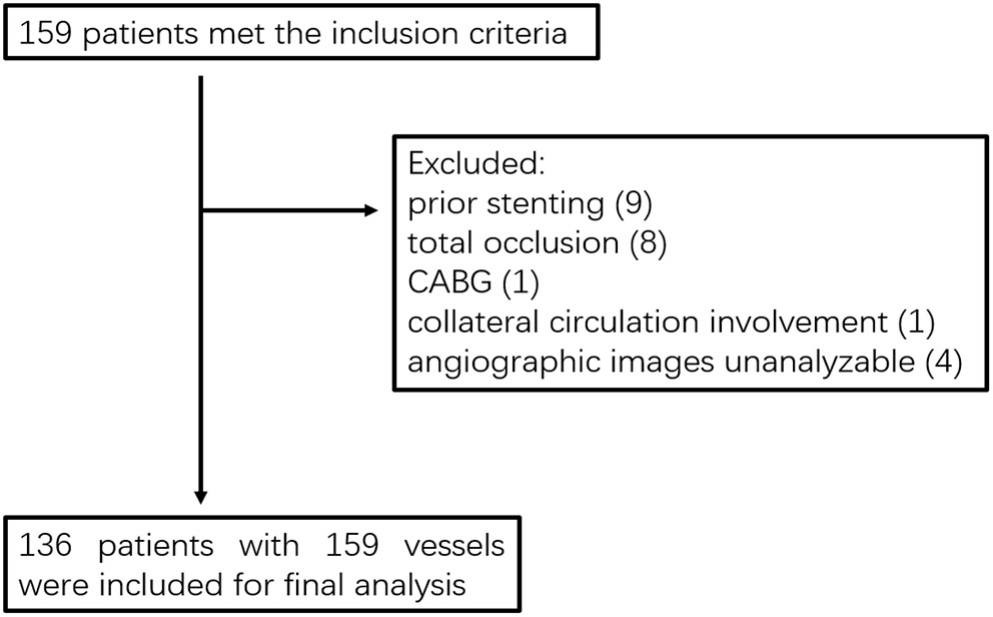
Study flowchart. CABG, coronary artery bypass grafting.

From April 2009 to June 2014, a total of 159 patients who were diagnosed with coronary artery disease with visual-determined significant coronary artery stenosis at the Department of Cardiology, Peking University First Hospital, participated in DES clinical trials for DES implantation. After screening according to inclusion and exclusion criteria (Figure 1), 136 patients with 159 vessels were included for further analysis. The baseline characteristics of the study population are reported in Table 1. The average age of patients was 59.01 ± 9.73, with 91 (66.91%) males and 45 (33.09%) females. Unstable angina was diagnosed in the majority of the patients (80.15%). The baseline lesion characteristics and details of the study procedure were shown in Table 2. The mean pre-PCI %DS and mean pre-PCI caFFR was 62.79 ± 17.53% and 0.63 ± 0.17, respectively, and the locations of the lesions included the left anterior descending artery (LAD, 56.60%), left circumflex artery (LCX, 20.76%), and right coronary artery (RCA, 22.64%).

**Table 1:** Baseline characteristics of study patients (*N* = 136).

	**Total (*N =* 136)**	**caFFR <0.9 (*N =* 49)**	**caFFR≥0.9 (*N =* 87)**	***P* value**
Age	59.01 ± 9.73	59.98 ± 9.60	58.46 ± 9.82	0.384
Male	91 (66.91%)	27 (55.10%)	64 (73.56%)	0.028
BMI	25.97 ± 3.73	26.46 ± 3.29	25.69 ± 3.95	0.273
Current smokers	53 (39.87%)	17 (34.69%)	36 (41.38%)	0.443
Current alcohol intake	31 (22.79%)	8 (16.33%)	23 (26.44%)	0.415
Hypertension	99 (72.79%)	34 (69.39%)	65 (74.71%)	0.501
Diabetes mellitus	41 (30.15%)	17 (34.69%)	24 (27.59%)	0.386
LDL-C	2.26 ± 0.68	2.36 ± 0.76	2.21 ± 0.63	0.227
CREA	86.68 ± 19.39	77.63 ± 16.97	83.96 ± 20.37	0.068
BNP	68.22 (34.50–177.22)	105.87 (36.00–214.80)	55.500 (33.63–138.75)	0.243
LVEF	66.04 ± 11.45	63.66 ± 13.25	67.39 ± 10.13	0.071
Prior MI	16(11.76%)	9 (18.37%)	7 (8.05%)	0.073
Prior PCI	7 (5.15%)	4 (8.16%)	3 (3.45%)	0.232
**Number of diseased vessel(s)**				0.033
1	70 (51.47%)	19 (38.78%)	51 (58.62%)	
2	43 (31.62%)	17 (34.69%)	26 (29.89%)	
3	23 (16.91%)	13 (26.53%)	10 (11.49%)	

*Values are n (%), mean±SD or median (IQR). SD, standard deviation; IQR, interquartile range; caFFR, coronary angiography-derived fractional flow reserve; BMI, body mass index; LDL-C, low-density lipoprotein cholesterol; CREA, creatinine; BNP, type B natriuretic peptide; LVEF, left ventricular ejection fraction; MI, myocardial infarction; PCI, percutaneous coronary intervention*.

**Table 2:** Pre- and Post-procedural characteristics of study vessels (*N* = 159).

	**Total (*N =* 159)**	**caFFR <0.9 (*N =* 57)**	**caFFR ≥0.9 (*N =* 102)**	***P* value**
**Location of lesion**				0.057
LAD	90 (56.60%)	39 (68.42%)	51 (50.00%)	
LCX	33 (20.76%)	7 (12.28%)	26 (25.49%)	
RCA	36 (22.64%)	11 (19.30%)	25 (24.51%)	
**Pre-procedural**				
Diameter stenosis, %	62.79 ± 17.53	64.19 ± 16.83	62.01 ±17.95	0.453
Minimal luminal diameter, mm	1.05 ± 0.51	0.92 (0.74–1.22)	1.09 (0.63–1.52)	0.181
Reference luminal diameter, mm	2.85 ± 0.52	2.76 ± 0.49	2.90 ± 0.53	0.102
Lesion length, mm	14.24 (9.14–23.25)	14.93(10.74–29.27)	13.70 (8.60–22.21)	0.096
caFFR	0.63 ± 0.17	0.59 ± 0.17	0.65 ± 0.17	0.042
Trans-lesion caFFR gradient	0.29 (0.19–0.44)	0.30 (0.21–0.46)	0.27 (0.15–0.41)	0.096
**Post-procedural**				
Residual stenosis, %	12.00 (7.50–15.00)	12.00 (9.00–15.00)	11.00 (7.00–15.00)	0.447
Minimal luminal diameter, mm	2.55 ± 0.41	2.52 ± 0.42	2.57 ± 0.40	0.429
**Number of stents**				0.104
1	129 (81.1%)	43 (75.4%)	86 (84.3%)	
2	28 (17.6%)	12 (21.1%)	16 (15.7%)	
3	2 (1.3%)	2 (3.5%)	0 (0%)	
Stent diameter, mm	2.96 ± 0.42	2.86 ± 0.36	3.03 ± 0.44	0.006
Stent length, mm	25.59 ± 10.33	26.14 ± 12.14	25.28 ± 9.22	0.618
caFFR	0.90 ± 0.06	0.84 ± 0.07	0.94 ± 0.02	<0.001
ΔcaFFR_stent_	0.04 (0.02–0.08)	0.09 (0.06–0.12)	0.04 (0.02–0.05)	<0.001
ΔcaFFR _stent_=0	12 (7.55%)	6 (10.53%)	6 (5.88%)	
ΔcaFFR _stent_>0	147 (92.45%)	51 (89.47%)	96 (94.12%)	
ΔcaFFR _stent/length_	0.02 (0.01–0.03)	0.03 (0.01–0.05)	0.01 (0.01–0.02)	<0.001

*Values are n (%), mean±SD or median (IQR). SD, standard deviation; IQR, interquartile range; LAD, left anterior descending artery; LCX, left circumflex artery; RCA, right coronary artery; caFFR, coronary angiography-derived fractional flow reserve; ΔcaFFRstent, caFFR gradient across stent; ΔcaFFRstent/length, caFFR gradient across stent divided by stent length and multiple by 10*.

### Post-PCI Functional Assessments of Target Vessels

Post-PCI caFFR and trans-stent caFFR gradient were obtained through coronary angiography performed immediately after stenting. The mean post-PCI caFFR was 0.90 ± 0.06. The median ΔcaFFR_stent_ was 0.04 (interquartile range 0.02–008). ΔcaFFR_stent_ > 0 was demonstrated in 147 vessels (92.45%). Taking the stent length into consideration, the median ΔcaFFR_stent/length_ was 0.02 (interquartile range 0.01–0.03).

To investigate the correlation between post-PCI functional indexes and baseline characteristics, caFFR = 0.90 was used as a cut-off value to stratify the study population (12, 17), there were more females, lower ratio of multivessel disease, lower pre-PCI caFFR value and lower stent diameter in the post-PCI caFFR <0.90 subgroup, with statistical significance (Tables 1, 2). When stratified by ΔcaFFR_stent_ (cut-off value 0.04) or ΔcaFFR_stent/length_ (cut-off value 0.009) (17), significant differences were found between the groups in gender, diabetes mellitus, location of lesion, reference vessel diameter, baseline CREA level, and stent length (Supplementary Tables 1, 2).

### Clinical Follow-Up and QCA at 9 Months

All enrolled patients completed clinical follow-up at 9 months. Among them, 130 patients with 152 vessels also completed a coronary angiography at the follow-up time. The follow-up QCA measurements and clinical events are presented in Tables 3, 4. The mean %DS at 9-month follow-up was 14.00 (9.00–19.25)%, and the mean MLD at follow-up was 2.48 ± 0.44 mm, with a LLL of 0.04 (−0.05–0.16) mm. In total 59.12% of vessels had LLL. For the 9-month follow-up events, 5 (3.68%) patients had TVF, of which 3 (3.22%) had TVR, 2 (1.47%) had cardiac death. in addition, 8 (5.88%) had non-target vessel revascularization and 1 (0.47%) had non-cardiac death.

**Table 3:** Quantitative coronary angiography at 9-month follow-up.

		**Classified by caFFR**			**Classified by caFFR**_**stent**_			**Classified by caFFR**_**stent/length**_		
	**Total** **(*N =* 159)**	**caFFR <0.9 (*N =* 57)**	**caFFR ≥0.9 (*N =* 102)**	***P* value**	**ΔcaFFR_**stent**_ <0.04** **(*N =* 61)**	**ΔcaFFR_**stent**_ ≥0.04** **(*N =* 98)**	***P* value**	**ΔcaFFR_**stent/length**_ <0.009** **(*N =* 38)**	**ΔcaFFR_**stent/length**_ ≥0.009** **(*N =* 121)**	***P* value**
%DS, %	14.00 (9.00–19.25)	15.00 (11.00–20.00)	13.00 (9.00–19.00)	0.159	14.00 (10.50–18.00)	14.00 (9.00–20.00)	0.533	14.00 (12.00–19.00)	14.00 (9.00–19.50)	0.609
MLD, mm	2.48 ± 0.44	2.42 ± 0.44	2.52 ± 0.43	0.191	2.48 ± 0.47	2.48 ± 0.41	0.972	2.45 ± 0.49	2.49 ± 0.42	0.570
LLL, mm	0.04 (−0.05–0.16)	0.04 (−0.02–0.18)	0.05 (−0.07–0.14)	0.464	0.02 (−0.05–0.14)	0.05 (−0.04–0.18)	0.606	0.02 (−0.05–0.11)	0.05 (−0.03–0.18)	0.313

*Values are mean±SD or median (IQR). SD, standard deviation; IQR, interquartile range; %DS, percent diameter stenosis; MLD, minimal lumen diameter; LLL, late lumen loss; caFFR, coronary angiography-derived fractional flow reserve; ΔcaFFRstent, caFFR gradient across stent; ΔcaFFRstent/length, caFFR gradient across stent divided by stent length and multiple by 10*.

**Table 4:** Clinical events at 9-month follow-up.

		**Classified by caFFR**			**Classified by caFFR**_**stent**_			**Classified by caFFR**_**stent/length**_		
	**Total** **(*N =* 136)**	**caFFR <0.9 (*N =* 49)**	**caFFR ≥0.9 (*N =* 87)**	***P* value**	**ΔcaFFR_**stent**_ <0.04** **(*N =* 47)**	**ΔcaFFR_**stent**_ ≥0.04** **(*N =* 89)**	***P* value**	**ΔcaFFR_**stent/length**_ <0.009** **(*N =* 26)**	**ΔcaFFR_**stent/length**_ ≥ 0.009** **(*N =* 110)**	***P* value**
TVR	3 (2.21%)	3 (6.12%)	0 (0.00%)	0.020	0 (0.00%)	3 (3.37%)	0.203	0 (0.00%)	3 (2.73%)	0.394
Non-target vessel revascularization	8 (5.88%)	4 (8.16%)	4 (4.60%)	0.396	5 (10.64%)	3 (3.37%)	0.124	4 (15.38%)	4 (3.64)	0.043
Cardiac death	2 (1.47%)	1 (2.04%)	1 (1.15%)	0.678	1 (2.13%)	1 (1.12%)	0.644	1 (3.85%)	1 (0.91%)	0.263
Non-cardiac death	1 (0.74%)	0 (0%)	1 (1.15%)	0.992	0 (0.00%)	1 (1.12%)	1.000	0 (0.00%)	1 (0.91%)	1.000
TVF	5 (3.68%)	4 (8.16%)	1 (1.15%)	0.037	1 (2.13%)	4 (4.49%)	0.485	1 (3.85%)	4 (3.64%)	0.959

*Values are n (%). TVR, target vessel revascularization; TVF, target vessel failure; caFFR, coronary angiography-derived fractional flow reserve; ΔcaFFR_stent_, caFFR gradient across stent; ΔcaFFR_stent/length_, caFFR gradient across stent divided by stent length and multiple by 10*.

We found no significant difference in the QCA results at follow-up between the subgroups stratified by post-PCI caFFR of 0.90, ΔcaFFR_stent_ of 0.04 or ΔcaFFR_stent/length_ of 0.009 (Table 3). However, the TVF rate was significantly higher in patients with post-PCI caFFR <0.90 compared with patients with optimal post-PCI caFFR (≥0.90) (4 [8.16%] vs. 1 [1.15%], *P* = 0.037), which was mainly caused by the difference in TVR (Table 4). The details of the post-PCI caFFR and trans-stent caFFR gradient of the 5 patients with TVF are presented in Table 5.

**Table 5:** Post- percutaneous coronary intervention (PCI) functional assessments of 5 patients with target vessel failure (TVF) during 9-month follow-up time.

**Patient**	**Age**	**Sex**	**Target vessel**	**Event**	**caFFR**	**ΔcaFFR_**stent**_**	**ΔcaFFR_**stent/length**_**
1	73	Male	LAD	Target vessel revascularization	0.78	0.18	0.042
2	74	Male	LAD	Target vessel revascularization	0.89	0.10	0.071
3	49	Female	LAD	Target vessel revascularization	0.88	0.09	0.021
4	70	Female	RCA	Cardiac death	0.84	0.10	0.022
5	73	Male	LAD	Cardiac death	0.91	0.01	0.003

*LAD, left anterior descending artery; RCA, right coronary artery; caFFR, coronary angiography-derived fractional flow reserve; ΔcaFFR_stent_, caFFR gradient across stent; ΔcaFFR_stent/length_, caFFR gradient across stent divided by stent length and multiple by 10*.

### Predictive Values of the Post-PCI CaFFR and Trans-stent CaFFR Gradient for Angiographic and Clinical Outcomes

To investigate the predictive role of post-PCI caFFR and trans-stent caFFR gradient on patient prognosis, we used univariate and multivariate linear regression models to explore the predictive role of post-PCI functional indicators on follow-up QCA measurements and used one-way logistic regression to find the predictive role of post-PCI functional assessments on follow-up clinical events. We did not find a statistically significant predictive value of post-PCI caFFR or trans-stent caFFR gradient for LLL, follow-up %DS and follow-up clinical events, either univariately or after adjusting for age, sex, and diabetes mellitus (Table 6). Subgroup analysis were then performed based on the location of the stents (Table 6) which revealed that post-PCI caFFR was predictive for LLL and follow-up %DS in the subgroup with lesions located in the LAD, and ΔcaFFR_stent_ was also predictive for LLL in the LAD subgroup. For every 1-unit increase in target vessel post-PCI caFFR, LLL was reduced by 1.07 mm (β = −1.07, 95% CI: −1.74 to −0.39, *P* = 0.002) and the %DS at follow-up was reduced by 30.24% (β = −30.24, 95% CI: −56.44 to −4.04, *P* = 0.025). LLL increased by 0.98 mm for each 1-unit increase in ΔcaFFR_stent_ (β = 0.98, 95% CI:0.13–1.83, *P* = 0.026) (Table 6).

**Table 6:** Predictive value of post-PCI coronary angiography-derived fractional flow reserve (caFFR), ¬caFFR gradient across stent (ΔcaFFR_stent)_, and¬caFFR gradient across stent divided by stent length and multiple by 10 (ΔcaFFR_stent/length)_ for late lumen loss (LLL) and percent diameter stenosis (%DS) at 9-month follow-up.

	**Univariate**		**Multivariate model^†^**		**LAD subgroup^‡^**	
	**β (95% CI)**	***P* value**	**β (95% CI)**	***P* value**	**β (95% CI)**	***P* value**
**LLL**						
caFFR	−0.31 (0.82,0.20)	0.240	−0.30 (−0.81,0.21)	0.260	−1.07 (−1.74,−0.39)	0.002
ΔcaFFR_stent_	0.61 (−0.14,1.35)	0.110	0.63 (−0.11, 1.37)	0.098	0.98 (0.13,1.83)	0.026
ΔcaFFR_stent/length_	0.65 (−0.54,1.84)	0.290	0.69 (−0.49, 1.87)	0.250	0.04 (−3.39,3.48)	0.980
**%DS**						
caFFR	−11.41 (−31.42,8.60)	0.270	−10.05 (−29.99,9.89)	0.320	−30.24 (−56.44,−4.04)	0.025
ΔcaFFR_stent_	5.29 (−24.01, 34.58)	0.720	3.03 (−25.97,32.04)	0.830	17.53 (−15.43,50.49)	0.290
ΔcaFFR_stent/length_	−14.79 (−61.47,31.89)	0.540	−17.66 (−63.60,28.29)	0.450	−31.37 (−163.52,100.78)	0.640

† *Multivariate model adjusting for age, gender and diabetes mellitus*.

‡ *Multivariate model adjusting for age, gender and diabetes mellitus applied to the subgroup with lesions located in LAD. 95% CI, 95% confidence interval; LLL, late lumen loss; %DS, percent diameter stenosis; LAD, center anterior descending artery; caFFR, coronary angiography-derived fractional flow reserve; ΔcaFFR_stent_, caFFR gradient across stent; ΔcaFFR_stent/length_, caFFR gradient across stent divided by stent length and multiple by 10*.

## Discussion

This study is a retrospective study conducted to investigate the predictive value of post-PCI caFFR and trans-stent caFFR gradient for neointimal proliferation of the target vessel and adverse events after a successful PCI. We chose patients who participated in DES clinical trials as the study population to minimize potential confounding factors as their baseline characteristics and medical treatments were more unified. All the measurements and calculations in this study were performed offline by two independent operators and each operator was required to repeat these measurements twice at two different times separated by at least 1 month to ensure the consistency of measurement and the veracity of data. The main findings of current study are as follows:

(1) Suboptimal caFFR (<0.90) was common among vessels immediately after angiographically satisfactory PCI with stent implantation; (2) a caFFR gradient across the stent was presented in more than 90% of vessels immediately after stenting; (3) suboptimal post-PCI caFFR was associated with adverse clinical events at 9-month follow-up; (4) both post-PCI caFFR and trans-stent caFFR gradient were independent predictive factors of neointimal proliferation (evaluated by LLL and %DS at follow-up) when the lesion was located in the left anterior descending coronary artery.

### Coronary Physiology Immediately After PCI

Despite an angiographically satisfactory PCI, many studies have demonstrated that impaired coronary physiology, expressed by suboptimal post-PCI FFR (9, 10, 12, 17, 29) or FFR related index (25), was found in significant proportion of vessels immediately after stenting. Although the definition of suboptimal post-PCI FFR varied in those studies, ranging from ≤ 0.80 to <0.90 based on the latest researches (12, 17), we chose 0.90 as the cutoff value to define suboptimal post-PCI caFFR. Consistent with previous studies, suboptimal post-PCI caFFR was presented in 35.8% of the studied vessels from 36.0% of patients. In the HAWKEYE study, researchers found LAD location, lesion length and post-PCI %DS as significant predictors of a lower QFR (a kind of angiography-derived FFR) (25). In the current study, we did not find statistically significant differences in the lesion location, lesion length, and post-PCI %DS between vessels with optimal post-PCI caFFR and vessels with suboptimal post-PCI caFFR, although a trend of higher ratio of LAD location could be observed in vessels with suboptimal post-PCI caFFR (68.42 vs. 50.00%, *P* = 0.057) (Table 2).

Using intravascular ultrasound (IVUS), Zandvoort and colleagues investigated 100 vessels with post-PCI FFR ≤ 0.85 and found that focal lesions, stent underexpansion, and malapposition in vessels may account for the dissatisfaction of post-PCI FFR, which were not readily apparent on angiography (8). Through the analysis of the localization of the QFR drop, Biscaglia and colleagues also drew a similar conclusion and added diffuse disease as another reason of impaired coronary physiology immediately after PCI. For stent underexpansion, a recent study had confirmed two indexes reflecting FFR gradient across stent (ΔFFR_stent_, ΔFFR_stent/length_) as indicators of suboptimal stent expansion assessed by IVUS and found that ΔFFR_stent_ > 0 was a common phenomenon in vessels immediately after stenting (17). As is shown in Table 2, ΔcaFFR_stent_>0 was also demonstrated in majority of the investigated vessels in our study. What's more, both ΔcaFFR_stent_ and ΔcaFFR_stent/length_ were significantly higher in vessels with post-PCI caFFR <0.90, supporting stent underexpansion as a reason counts for suboptimal functional indexes after PCI.

### Prognostic Predictive Value of Post-PCI CaFFR and Trans-stent CaFFR Gradient

The prognostic predictive value of the post-PCI functional assessment was investigated by many studies with various endpoints and different follow-up times (9–12, 25, 29). As a consensus of those literatures, suboptimal post-PCI FFR (wire-based or angiography-derived) can be an independent predictor of target vessel related events. In current study, using 0.90 as the cutoff value, we found a correlation between suboptimal post-PCI caFFR and TVF at 9-month follow-up (Table 4), but in the multivariate analysis, we failed to identify post-PCI caFFR as an independent predictor of TVF (Supplementary Table 3). That may be accounted by the lower rate of adverse events in the 9-month follow-up time. As for trans-stent FFR gradient, a recent study detected both ΔFFR_stent_ and ΔFFR_stent/length_ as predictors of MACE during a 10-year follow-up time (17). There is no previous study on ΔcaFFR_stent_ or ΔcaFFR_stent/length_, and our research reported the result for the first time. However, we haven't found a significant association between ΔcaFFR_stent_ or ΔcaFFR_stent/length_ and 9-month TVF in the total study population (Table 4), although suboptimal trans-stent gradient was seen in majority of patients with 9-month TVF (Table 5).

In the analysis of coronary angiography data at follow-up time using multivariate regression models, we found that post-PCI caFFR was an independent predictor of LLL and %DS at follow-up, and ΔcaFFR_stent_ was an independent predictor of LLL in lesions with LAD location (Table 6). In previous studies, LAD location was found to be associated with worse post-PCI functional indexes and higher TVF rate (9, 25), which was possibly because of a large territory of myocardium in LAD perfusion, making any stenosis in LAD have a larger impact on coronary physiology (9). In the current study, we also found a statistically significant higher rate of LAD location in vessels with ΔcaFFR_stent_ ≥ 0.04 (64.29 vs. 44.26%, *P* = 0.038, Supplementary Table 2), further revealing the correlation between LAD location and suboptimal coronary physiology immediately after stenting. The results of regression analysis, combined with this finding, indicated the values of post-PCI caFFR and caFFR gradient across stent in the prediction of neointimal proliferation of LAD stenting, although the optimal cutoff values and the possibility of applying these indexes to lesions located in LCX or RCA still need further investigation.

### Study Limitation

This study was a retrospective study with its inherent limitations. Although majority of the baseline characteristics were quite comparable among patients with optimal post-PCI functional assessments and patients with suboptimal post-PCI functional assessments, there were still some characteristics like gender, diabetes mellitus, and the number of diseased vessels showing significant differences between groups. This study was conducted in a single center, which could make the treatments and follow-up more standardized. However, the patient population in a single center may be a lack of representation. Last but not least, the limited number of events due to the small sample size was also a main limitation of our study and the 9-month follow-up time may be not sufficient to detect enough events and revealed the value of the indexes we studied in the prediction of long-term clinical outcomes. Despite these limitations, the current study initially and preliminarily demonstrated post-PCI caFFR and its gradient across stent can be reliable assessments of coronary physiology immediately after stenting and potential predictors of restenosis of treated vessels and patients' prognosis. Based on this inspiring finding, another study with enlarged sample size and prolonged follow-up time is on-going in our center. Further, prospective multicenter studies are also needed.

## Conclusion

The measurement of caFFR immediately after PCI with DES implantation is feasible. Suboptimal post-PCI caFFR was demonstrated in significant proportion of vessels and was associated with a higher rate of TVF. When the lesion was located in the LAD, caFFR could be an independent predictor of LLL and %DS at follow-up. Trans-stent caFFR gradient existed in more than 90% of the treated vessels, and it was also an independent predictor of LLL with lesions located in LAD.

## Data Availability Statement

The original contributions presented in the study are included in the article/Supplementary Materials, further inquiries can be directed to the corresponding author/s.

## Ethics Statement

The studies involving human participants were reviewed and approved by Peking University First Hospital Human Research Ethics Committee. Written informed consent for participation was not required for this study in accordance with the national legislation and the institutional requirements.

## Author Contributions

ZZ wrote and edited the manuscript. BZ assessed the patients for study eligibility, collected data, participated in writing the manuscript. FF performed data analysis. FY and YG performed QCA and caFFR measurements. SF, ZW, and LQ helped in data collection and patient follow-up. YG and YH designed the study, helped in editing the manuscript. All authors contributed significantly to this work and supported the publication of the manuscript. All authors contributed to the article and approved the submitted version.

## Conflict of Interest

The authors declare that the research was conducted in the absence of any commercial or financial relationships that could be construed as a potential conflict of interest.

## Publisher's Note

All claims expressed in this article are solely those of the authors and do not necessarily represent those of their affiliated organizations, or those of the publisher, the editors and the reviewers. Any product that may be evaluated in this article, or claim that may be made by its manufacturer, is not guaranteed or endorsed by the publisher.
