# Supplementary material for: Prognostic Value of Coronary Angiography-Derived Fractional Flow Reserve Immediately After Stenting
Source: Front Cardiovasc Med. 2022 Mar 21;9:834553. doi: 10.3389/fcvm.2022.834553 (PMC8978525; doi:10.3389/fcvm.2022.834553)
Supplement: Supplementary file 1 [file Data_Sheet_1.pdf]

Supplemental Table 1: Baseline characteristics of study patients stratified by $\Delta caFFR_{stent}$ or $\Delta caFFR_{stent/length}$

	Total (N=136)	$\Delta caFFR_{stent}$ <0.04 (N=47)	$\Delta caFFR_{stent}$ ≥0.04 (N=89)	P value	$\Delta caFFR_{stent/length}$ <0.009 (N=26)	$\Delta caFFR_{stent/length}$ ≥0.009 (N=110)	P value
Age	59.01±9.73	57.83±9.56	59.63±9.82	0.307	56.62±10.65	59.57±9.47	0.164
Male	91 (66.91%)	37 (78.72%)	54 (60.67%)	0.033	20 (76.92%)	71 (64.55%)	0.228
BMI	25.97±3.73	26.27±4.21	25.81±3.47	0.515	26.49±4.89	25.85±3.45	0.466
Current smokers	53 (39.87%)	19 (40.43%)	34 (38.20%)	0.800	9 (34.62%)	44 (40.00%)	0.613
Current alcohol intake	31 (22.79%)	14 (29.79%)	17 (19.10%)	0.158	6 (23.08%)	25 (22.73%)	0.970
Hypertension	99 (72.79%)	33 (70.21%)	66 (74.16%)	0.623	17 (65.38%)	82 (74.55%)	0.345
Diabetes mellitus	41 (30.15%)	9 (19.15%)	32 (35.96%)	0.042	5 (19.23%)	36 (32.73%)	0.177
LDL-C	2.26±0.68	2.22±0.64	2.29±0.70	0.560	2.33±0.65	2.25±0.69	0.589
CREA	86.68±19.39	85.75±21.94	79.53±17.66	0.075	88.39±26.77	80.09±16.97	0.049
BNP	68.22 (34.50-177.22)	48.79(32.50-138.00)	78.13 (40.95-207.97)	0.092	36.00 (29.00-68.22)	78.13 (39.50-190.12)	0.060
LVEF	66.04±11.45	67.61±10.22	65.22±12.02	0.253	66.32±11.09	65.98±11.58	0.894
Prior MI	16(11.76%)	4 (8.51%)	12 (13.48%)	0.392	2 (7.69%)	14 (12.73%)	0.474
Prior PCI	7 (5.15%)	2 (4.26%)	5 (5.62%)	0.732	2 (7.69%)	5 (4.55%)	0.618
Number of diseased vessel(s)				0.636			0.957
1	70 (51.47%)	25 (53.19%)	45 (50.56%)		14 (53.85%)	56 (50.91%)	
2	43 (31.62%)	16 (34.04%)	27 (30.34%)		8 (30.77%)	35 (31.82%)	
3	23 (16.91%)	6 (12.77%)	17 (19.10%)		4 (15.38%)	19 (17.27%)	

Values are *n* (%), mean±SD or median (IQR). SD, standard deviation; IQR, interquartile range; caFFR, coronary angiography-derived fractional flow reserve; BMI, body mass index; LDL-C, low-density lipoprotein cholesterol; CREA, creatinine; BNP, type B natriuretic peptide; LVEF, left ventricular ejection fraction; MI, myocardial infarction; PCI, percutaneous coronary intervention.

Supplemental Table 2: Pre- and Post-procedural characteristics of study vessels stratified by $\Delta caFFR_{stent}$ or $\Delta caFFR_{stent/length}$

	Total (N=159)	$\Delta caFFR_{stent}$ <0.04 (N=61)	$\Delta caFFR_{stent}$ ≥0.04 (N=98)	P value	$\Delta caFFR_{stent/length}$ <0.009 (N=38)	$\Delta caFFR_{stent/length}$ ≥0.009 (N=121)	P value
Location of lesion				0.038			0.140
LAD	90 (56.60%)	27 (44.26%)	63 (64.29%)		19 (50.00%)	71 (58.68%)	
LCX	33 (20.76%)	15 (24.59%)	18 (18.37%)		6 (15.79%)	27 (22.31%)	
RCA	36 (22.64%)	19 (31.15%)	17 (17.35%)		13 (34.21%)	23 (19.01%)	
Pre-procedural							
Diameter stenosis, %	62.79±17.53	62.88±17.17	62.73±17.85	0.958	65.78±16.52	61.85±17.80	0.228
Minimal luminal diameter, mm	1.05±0.51	1.08 (0.67-1.62)	1.00 (0.64-1.34)	0.311	0.97 (0.60-1.60)	1.00 (0.68-1.43)	0.883
Reference luminal diameter, mm	2.85±0.52	2.97±0.57	2.78±0.47	0.022	3.00±0.59	2.81±0.49	0.052
Lesion length, mm	14.24 (9.14-23.25)	13.15 (9.10-22.18)	14.59 (9.19-24.55)	0.394	16.84 (10.88-27.00)	14.16 (8.90-22.48)	0.081
caFFR	0.63±0.17	0.64±0.18	0.63±0.16	0.640	0.59±0.18	0.65±0.17	0.092
Trans-lesion caFFR gradient	0.29 (0.19-0.44)	0.26 (0.15-0.39)	0.32 (0.19-0.44)	0.139	0.28 (0.21-0.44)	0.29 (0.18-0.43)	0.594
Post-procedural							
Residual stenosis, %	12.00 (7.50-15.00)	12.00 (8.00-16.00)	11.00 (7.00-15.00)	0.371	13.50 (8.25-18.25)	11.00 (7.00-15.00)	0.118
Minimal luminal diameter, mm	2.55±0.41	2.54±0.45	2.56±0.38	0.774	2.48±0.44	2.57±0.40	0.226
Stent length, mm	25.59±10.33	25.50±10.08	25.64±10.54	0.937	29.00±11.18	24.52±9.86	0.019
caFFR	0.90±0.06	0.92±0.08	0.88±0.05	0.005	0.91±0.09	0.89±0.05	0.205
$\triangle caFFR_{stent}$	0.04 (0.02-0.08)				0.01 (0.00-0.020)	0.06 (0.04-0.09)	<0.001
$\triangle caFFR_{stent/length}$	0.02 (0.01-0.03)	0.01 (0.00-0.02)	0.02 (0.02-0.04)	<0.001			

Values are *n* (%), mean±SD or median (IQR). SD, standard deviation; IQR, interquartile range; LAD, left anterior

descending artery; LCX, left circumflex artery; RCA, right coronary artery; caFFR, coronary angiography-derived fractional flow reserve; $\triangle caFFR_{stent}$, caFFR gradient across stent; $\triangle caFFR_{stent/length}$, caFFR gradient across stent divided by stent length and multiple by 10.

Supplemental Table 3: Predictive value of post PCI caFFR, $\Delta caFFR_{stent}$ and $\Delta caFFR_{stent/length}$ for TVF at 9-month follow-up

	Univariate		Multivariate model 1†		Multivariate model 2‡	
	OR (95%CI)	P value	OR (95%CI)	P value	OR (95%CI)	P value
caFFR	0.13 (0.01,1.20)	0.070	0.14 (0.01,1.34)	0.080	0.15 (0.01,1.42)	0.090
$\Delta caFFR_{stent}$	2.55 (0.28,23.38)	0.400	2.07 (0.21,20.35)	0.530	2.03 (0.20,20.53)	0.540
$\Delta caFFR_{stent/length}$	1.26 (0.14,11.67)	0.830	0.99 (0.10, 9.71)	0.990	1.05 (0.11,10.49)	0.960

†Multivariate model adjusting for age and gender. ‡Multivariate model adjusting for age, gender and diabetes mellitus. OR, odds ratio; 95%CI, 95% confidence interval; TVF, target vessel failure; caFFR, coronary angiography-derived fractional flow reserve; $\triangle caFFR_{stent}$, caFFR gradient across stent; $\triangle caFFR_{stent/length}$, caFFR gradient across stent divided by stent length and multiple by 10.
